# Categorizing errors in high‐reliability organizations: Adaptive range and adaptive capacity in incident response

**DOI:** 10.1111/risa.70024

**Published:** 2025-03-22

**Authors:** Elmar Kutsch, Haytham Siala, Chantal Cantarelli, Ibrat Djabbarov

**Affiliations:** ^1^ School of Management Cranfield University Cranfield UK; ^2^ The School of Business and Management Queen Mary University of London London UK; ^3^ Management and Entrepreneurship Department Imperial College Business School London UK

**Keywords:** accidents, errors, high‐reliability organizations, organizational response, repertory grid

## Abstract

This study examines how actors in a high‐reliability organization categorize errors as near‐misses or accidents through the lens of adaptive capacity and adaptive range. We studied a large defense entity with operations critical to national security to understand how organization members categorized errors during incidents. Using the repertory grid method to interview informants, we identify key dualities that actors navigate between anticipatory and retrospective responses to errors. These dualities collectively reflect the organization's adaptive capacity and adaptive range when balancing anticipatory and retrospective responses. Our analysis of error categorization through this lens provides new insights into how high‐reliability organizations manage incidents to maintain reliability and offers practical implications for enhancing organizational resilience in high‐risk settings.

## 1 INTRODUCTION

High‐reliability organizations (HROs) operate under constant pressure to prevent disastrous events (e.g., Roberts, 1990a, 1990b; Roberts & Bea, 2001; Weick & Sutcliffe, 2015; Weick et al., 1999). Effectively managing near‐misses and accidents is essential for HRO's survival. Organizations in industries such as nuclear energy, aviation, and defense are subject to extreme risks and require robust safety, incident management, and continuous vigilance approaches (Weick & Roberts, 1993). Scholars suggest a dual strategy combining anticipatory and retrospective responses to near‐misses and accidents (Dillon et al., 2014; Goodman et al., 2011; Hopkins, 2009b; Vogus et al., 2010). Such an approach promotes constant attention to continuous improvement, which is critical for maintaining operational integrity and safety in these high‐stakes environments (Snook, 2000).

Near‐miss errors, generally defined as unintentional and potentially preventable deviations from established goals or standards resulting in neutral outcomes (Hofman & Frese, 2011; Lei et al., 2016) are particularly significant to the functioning of HROs. Incidents offer organizations valuable, cost‐effective opportunities for anticipatory intervention. Near‐misses offer early warning signs (Weick & Sutcliffe, 2015), highlighting potential system vulnerabilities before they escalate into accident failures. As such, they provide organizations with critical insights into areas where protocols may need strengthening. In contrast, accidents involve negative consequences, sometimes leading to disastrous outcomes, as seen in events like the Chernobyl nuclear disaster (Plokhy, 2018) and the Bhopal plant explosion (Shastri, 2020; Vogus et al., 2010). Organizations adopting near‐miss reporting systems learn from minor adverse events. For instance, in hospitals, clinicians regularly conduct reviews of clinical mistakes to learn and introduce necessary changes to clinical processes or procedures.

However, strict protocols and safety measures do not fully safeguard organizations from mistakes. Accidents reveal vulnerabilities of the existing protocols, including aspects not previously noticed or anticipated in complex systems. In this sense, accidents can expose hidden systemic flaws even when rigorous incident management measures are in place. Unlike near‐miss errors, which offer anticipatory learning opportunities, accidents require retrospective learning. While near‐misses and accidents trigger distinct responses, our understanding of the nature and structure of these responses remains limited. Furthermore, how organization members interpret and assign meaning to their responses to incidents in pursuit of high reliability remains underexamined. As a result, this paper explores the cognitive frames that shape adaptive capacity and support adaptive responses to near‐misses and accidents.

The adaptive range refers to the spectrum of possible responses organization members can deploy in response to near‐miss errors and accidents. Adaptive capacity is the organization's ability to effectively and flexibly respond to incidents using adaptive range, drawing on cognitive flexibility to manage unexpected disruptions (Fraher et al., 2017). Rather than switching between distinct response poles, managers often navigate within this adaptive range, operating in the ambiguous space between extremes, where the boundaries and consequences are unclear. This study sheds light on how these cognitive dynamics influence early responses to near‐miss errors and accidents, shaping the organization's ability to maintain high reliability in complex and evolving environments.

We used the repertory grid technique (Jankowicz, 2004) to conduct in‐depth interviews with 103 managers across various departments in a major European defense organization. Repertory grid‐based interviews allowed for a structured and comparative analysis of informants’ constructs of incidents construed as near‐miss or accidents. This technique, rooted in personal construct theory, is designed to elicit and compare individual constructs ‐how informants perceive, interpret, and categorize experiences—by systematically analyzing the similarities and differences between various elements or situations. Furthermore, this method helps researchers systematically probe into shared patterns of informants’ cognitive processes when responding to incidents (Cornelius, 2015; Cowley et al., 2021). Our analysis reveals a spectrum of eight bipolar response categories, demonstrating how managers adapt their response strategies based on the perceived severity and implication of incidents.

Our study contributes to the literature on organizational responses to near‐misses and accidents by providing empirical evidence of how actors construe and adjust their actions to near‐miss and accidents. We identified eight categories of actions that constitute an adaptive range of HRO responses and how organization members use them to adjust their responses based on how they categorize incidents. By learning from near‐misses and effectively responding to accidents, organizations can create a comprehensive safety culture that continuously evolves, enhancing their overall resilience, operational integrity, and mindful decision‐making (Azadegan et al., 2019; Cooke & Rohleder, 2006; Dillon et al., 2016; Weick & Sutcliffe, 2015). This approach advocates for integrating the categorization of responses to near‐misses and accidents into a cohesive incident management framework. This framework can support proactive measures to prevent new incidents through anticipatory learning from near‐miss errors while ensuring reactive strategies are in place to address and learn from accidents as they arise.

## 2 LITERATURE BACKGROUND

### 2.1 The role of categorization and early response in managing near‐miss errors and accident failures in complex systems

Normal accident theory highlights that in complex systems where multiple and tightly coupled elements interact, accidents are inevitable and can escalate from seemingly minor incidents or near‐misses into disastrous failures (Perrow, 1999; Turner, 1976). This theory suggests that the interconnectedness and interdependencies within such systems inherently increase the potential for minor errors to cascade through the system, magnifying their effects and leading to major accidents (Weick, 1993). In contrast, research on HROs suggests that accidents are avoidable through vigilant management and timely intervention (Roberts & Bea, 2001). However, timely intervention requires learning from these incidents and promptly implementing effective corrective responses (Perrow, 1999). By responding early and carefully to near‐miss errors, managers can ensure that the knowledge gained from these events is used to prevent issues from escalating into accidents and accidents from developing into disasters (March et al., 1991; Perrow, 1999; Turner, 1976).

In tightly coupled and complex systems, such as those in industries like chemical processing (Perrow, 1999), the timely recognition and early activation of a response to near‐misses and accident failures is critical to preventing potentially disastrous escalations. These systems are characterized by intricate interdependencies, where a seemingly minor error can rapidly cascade through the system, causing a chain reaction of failures. Quickly detecting and reacting to early warning signs, such as near‐miss incidents, is essential to halting this escalatory process before it leads to disastrous outcomes.

An early response is an important line of defense in HROs, buying critical time to assess the situation and implement corrective measures (Cooke & Rohleder, 2006; S. Dekker, 2011; March et al., 1991). Delays in responding can allow even minor incidents to intensify, transforming into full‐scale accidents with far‐reaching consequences. An early activation of a response helps contain the problem at its root, preventing it from expanding beyond control and allowing time to protect human life, valuable resources, and the organization's reputation. Understanding how managers and organizational members react to near‐miss errors and accident failures—particularly in early activating a response within a tightly coupled system—is important in improving incident management in high‐stakes environments. The temporal aspect of this response is essential. Activating an early response allows organizations to intervene before a situation spirals, preventing a minor issue from escalating into a major crisis. This proactive approach aligns with the HRO principles, emphasizing vigilance and timely intervention as key strategies for maintaining reliability and avoiding disaster (Hopkins, 2009b).

One of the key aspects in activating an early response to near‐miss errors and accident failures, as found in the HRO literature, is the cognitive process of categorization (S. Dekker, 2011; Edmondson, 1996; Rasmussen, 1997; Weick & Sutcliffe, 2015). Categorization is instrumental in rapidly processing complex, uncertain, and often ambiguous information to make informed decisions. Categorizing aids in structuring and simplifying complex information, making the response to an incident manageable and actionable. As a fundamental cognitive function, this process involves grouping objects, ideas, or experiences into categories by identifying shared characteristics or similarities (Lamont & Molnár, 2002). In the context of this study, near‐miss errors and accident failures are different categories, yet they share common characteristics in how people may experience and respond to them (S. Dekker, 2004; Phimister et al., 2003; Turner, 1976).

Categorization serves as a springboard to corrective responses to near‐miss errors and accident failures. People categorize events and experiences to simplify complex situations into meaningful categories that help them make sense of their experience by bracketing their attention and directing action (Tinsley et al., 2012). How people categorize incidents as near‐misses or accidents can alter their assessment and actions toward them (Kahneman & Miller, 1986). Similarly, according to Toren et al. (2021), managerial perceptions and the anticipated severity of near‐miss errors and accident failures influence the initiation of response actions. Near‐miss incidents are often antecedents of accident failures. However, because they are not perceived as precursors, the responses to near‐miss errors may differ from those of actual accident failures. Moreover, Tinsley et al. (2012) show how having categorized near‐misses as “almost happened,” individuals were likelier to adopt a corrective action than when accidents were interpreted as near‐miss errors that did not occur.

### 2.2 Exploring the adaptive range and capacity to responding to near‐misses and accidents

Understanding how individuals and managers categorize near‐misses and accidents is essential for assessing their adaptive range and capacity to respond effectively to such events. Categorization influences not only the perception of risk but also the urgency and type of corrective actions. When managers categorize near‐miss errors as “almost accidents,” it affects their adaptive capacity in handling future unforeseen events.

#### 2.2.1 Adaptive range

Adaptive range refers to the spectrum of response options that managers consider salient—those they deem relevant, actionable, and appropriate—when addressing events construed as near‐miss errors and accident failures (S. Dekker, 2011; Grote, 2009; March et al., 1991; Oktem & Meel, 2008; Reason, 1997; Weick & Sutcliffe, 2015; Woods & Branlat, 2011).

However, a significant gap exists in understanding the full range of responses that managers and organizational members develop in shaping their strategies and actions. These responses, which include varied interpretations and meanings assigned to near‐misses and accidents, are crucial for comprehending *how* reliability is pursued. In that regard, a key critique of HRO research is its emphasis on observable practices and outcomes, often overlooking the subjective, cognitive processes that shape decision‐making (e.g., Dwyer et al., 2023). These constructed responses significantly influence the organization's approach to reliability. However, current research focuses on responses that align with established HRO characteristics, such as standardization and redundancy. This narrow focus risks neglecting other critical responses that may not fit these predefined categories, leaving a broader spectrum of interpretations and strategies underexplored (S. W. A. Dekker & Nyce, 2014; Sagan, 1993).

#### 2.2.2 Adaptive capacity

HRO research suggests that the ability to respond to unexpected events is a cornerstone of organizational resilience and reliability. An organization's adaptive capacity is central to its resilience, as it reflects the ability to adjust dynamically to incidents (Reason, 1997; Roberts & Bea, 2001; Woods, 2006). The key to effective response is not fixed, rigid action plans and range of responses but rather maintaining a wide range of responses that enable real‐time adjustments, thereby avoiding the trap of “either‐or” thinking (Farjoun, 2010). Instead, HROs thrive by engaging in “both‐and” thinking, where responses can be adjusted dynamically within the adaptive range of response categories as conditions evolve (Weick et al., 1999).

Rather than choosing between two extremes of a response, such as complete restraint or full intervention, the organization can respond along a spectrum within a single response category. For instance, in response to a near‐miss, an organization may initially take a cautious approach by increasing heeding and monitoring or issuing a warning. However, as more information becomes available, that response can escalate dynamically—perhaps by introducing stricter safety protocols or temporarily halting certain activities. This kind of response flexibility reflects mindfulness in decision‐making, where the organization remains aware of current realities and potential future developments, adapting as conditions shift (Hales & Chakravorty, 2016).

This capacity for dynamic, real‐time adjustment is essential in HROs because incidents are construed as near‐misses, and accidents are not uniform in nature and severity (Weick & Sutcliffe, 2015; Woods, 2006). A flexible, adaptive approach allows the organization to consider the unique context of incidents construed as near‐misses and accidents, adjusting the intensity and scope of the response as more is understood about the situation. In this way, the organization avoids the binary trap of thinking that “either” a problem is serious enough for immediate and drastic action “or” insignificant enough to be dismissed (Farjoun, 2010; Weick, 1995).

Furthermore, “both‐and thinking” is crucial in balancing proactive and reactive responses. Rather than choosing between either being proactive in preventing incidents or being reactive in addressing them after they occur, HROs do both simultaneously. After a near‐miss, for instance, the organization might introduce preventative measures such as enhanced training or procedural changes (proactive) while closely monitoring operations for signs of similar risks (reactive). This ensures that the organization addresses the current issue and strengthens its future resilience. In this way, mindfulness is applied to real‐time decision‐making, enabling simultaneous attention to both the immediate situation and its broader implications (Staber & Sydow, 2002; Weick, 1995; Weick & Sutcliffe, 2015; Woods & Cook, 2006).

This idea of dynamic response along a spectrum can be applied across different categories of organizational behavior. For example, in communication, the response does not have to be “either” open and transparent “or” reserved and cautious. Instead, communication strategies can evolve in real time, becoming more detailed and transparent as the situation unfolds. Similarly, regarding safety protocols, responses can move fluidly between routine procedures and heightened protocols without being locked into one extreme.

### 2.3 Contribution

While the adaptive range of HROs theoretically allows for various response categories to near‐misses and accidents, the existing literature does not fully clarify the spectrum of responses managers deem relevant in practice. There is a gap in understanding how organizational leaders can navigate and utilize the full range of this adaptive capacity (Staber & Sydow, 2002). This includes responding effectively across the spectrum—from rule‐following behaviors to more flexible, improvisational responses.

Specifically, we lack a clear picture of whether managers leverage the adaptive capacity necessary to exploit the full benefits of this response range. For instance, some incidents may require strict adherence to established rules and protocols, particularly in high‐risk situations where deviations could be dangerous. On the other hand, certain situations might call for improvisation and creative problem‐solving, where rigidly following the rules could limit the organization's ability to adapt to novel challenges or unexpected crises. Improvisation is part of an organization's adaptive capacity, where improvisation can prevent near‐miss errors from escalating into accident failures. This strategy is effective in situations with high uncertainty and potential risks that could lead to unpredictable accident failures. However, improvisation could also worsen the situation and is not effective or appropriate in all situations (Trotter et al., 2013).

The literature often assumes that managers can effortlessly navigate between the extreme poles of a response. However, they often operate in the ambiguous space between these extremes without knowing where those limits lie or how their decisions may trigger an early response in tightly coupled systems. Factors such as organizational culture, managerial experience, and the nature of the incident itself can influence whether a manager leans toward rule‐following or improvisation. In some organizations, there might be a tendency to over‐rely on established rules, even when flexibility is needed. Conversely, other organizations may overemphasize improvisation, leading to inconsistent practices that undermine safety protocols (Hollnagel, 2014; Laporte, 1996; Reason, 2000; Weick & Sutcliffe, 2015).

The practical implications of this study are important for organizations operating in high‐stakes environments such as health care, aviation, and railways (Busby, 2006; Caspi et al., 2023; Hopkins, 2009b; Vogus et al., 2010). When managers do not fully leverage their adaptive range (the variety of response categories) and adaptive capacity (the ability to shift on the spectrum of a response category), organizations face heightened risks of disastrous failures. In complex systems, real‐time adaptation is essential for maintaining reliability and preventing cascading failures.

To achieve this, organizations may benefit from going beyond mere adherence to predefined rules and best practices, which often promote rigidity and static responses. Instead, they must adopt a more agile and dynamic approach incorporating improvisation and adaptive structuration. Improvisation is crucial when pre‐established solutions are either unavailable or inadequate (Miner et al., 2001; Vera & Crossan, 2005; Weick, 1993). It allows managers and teams to act creatively, flexibly, and decisively, enabling them to navigate complex and rapidly changing environments without being constrained by rigid procedures. This ability to respond spontaneously and innovate under pressure is key to managing unforeseen challenges and preventing minor incidents from escalating into more severe outcomes.

However, the flexibility provided by improvisation may be complemented by adaptive structuration and institutionalizing adaptive capacity (Giddens, 1984; March, 1991; Miner & Mezias, 1996; Rochlin et al., 1998), ensuring that the organization's systems, structures, and rules continuously evolve based on real‐time experiences. As managers and teams improvise during near‐misses and accidents, they learn and contribute to reshaping the organization's formal structures. This process allows the organization to institutionalize new practices, integrating flexibility, adaptability, and mindful decision‐making into its core systems.

## 3 METHODS

### 3.1 The research setting and sample

The context for our empirical study is a large European defense organization operating within the framework of an HRO tasked with developing and delivering critical equipment to protect human lives. The organization's work is closely linked to other entities, crucial to accomplishing its high‐stakes goals. A core aspect of ensuring this mission‐critical equipment's reliability involves identifying, mitigating, and responding to near‐misses and accident failures. Failure to address these incidents carries the potential for significant operational disruptions, delays in delivery, breaches of safety and security protocols, reputational damage due to the organization's high‐profile status, and substantial financial costs.

This study context presents important nuances and deviations from more traditional HRO settings. First, while failures in the organization can be severe, they do not generally involve immediate threats to human life. This adds a layer of complexity to managing near‐misses and accidents, as the risk profile does not always invoke the same immediate urgency. However, the long‐term consequences regarding safety, performance, and reputational impact can be equally significant.

Second, a distinctive challenge in this environment is the separation between management and technical operations. Managers in this organization often lack direct authority and control over core technological processes, making decision‐making and incident management more complicated. As Roberts (2009) highlights, this separation can hinder the swift and effective handling of errors, which contrasts sharply with HRO environments where managers directly oversee critical operations. This lack of direct control necessitates an adaptive approach to incident management that considers the organization's unique structural and operational dynamics and emphasizes coordination, communication, and leadership across functional boundaries.

In this respect, middle managers are a crucial link between senior leadership and frontline operations, bridging communication gaps, coordinating responses, and ensuring the smooth flow of information and decisions during critical incidents (Vogus & Sutcliffe, 2012). Although they often lack direct control over technical processes, middle managers contribute significantly to incident response efforts, providing leadership and decision‐making support (Harding et al., 2014; Quy Nguyen, 2001; Vogus & Sutcliffe, 2012). Their ability to coordinate, communicate, and facilitate effective responses underpins the organization's capacity to manage complexity and maintain high reliability.

We interviewed 103 middle managers in project and program management roles to understand how they categorize their responses to near‐misses and accidents. All participating managers in this study had at least ten years of experience, ensuring they had sufficient exposure to near‐miss errors and accident failures. The interviewees were asked to recall at least six notable incidents from different projects, enabling the study to draw insights from diverse perspectives and experiences. By doing so, we aimed to capture how these managers categorize and interpret near‐misses and accidents, informing how they activate and manage responses within a complex, tightly coupled organizational structure.

### 3.2 Repertory grid procedure

Repertory grid interviews are a methodological tool used primarily to uncover how individuals perceive and interpret their world through personal constructs. These constructs are unique cognitive frameworks each person uses to make sense of their experiences. In repertory grid interviews, the informants’ specific background or contextual details are generally considered nonessential. The technique is designed to extract personal constructs regardless of the respondent's circumstances, focusing instead on understanding them. The three main procedures in administering the repertory grid are the choice of elements, the elicitation of constructs, and the rating of the elements given the respondents’ constructs (Wright & Cheung, 2005).

#### 3.2.1 The choice of elements

We asked the respondents to select six memorable events they had actively managed, including three near‐misses and three accidents. To help them differentiate between these two categories, we provided general descriptions of near‐misses and accidents based on definitions from Dillon and Tinsley (2008) and Phimister et al. (2003).

- “Near‐miss: Unplanned event that had the *potential to cause, but did not result* in human injury, environmental or equipment damage, an interruption to normal operation, or the failure to meet project and program objectives.”
- “Accident: Unplanned event *that resulted* in human injury, environmental or equipment damage, an interruption to normal operation, or the failure to meet project and program objectives.”

The elements identified by participants ranged from workplace incidents such as trips and falls to non–life‐threatening consequences, delays, financial losses, quality defects, rework, and productivity inefficiencies. The broad spectrum of consequences of these near‐misses and accidents reflects real‐world incidents’ complex and multifaceted nature. The events are positioned as “probes” into individuals’ cognitive structures precisely because of their varied nature. This allows for a comprehensive exploration of how individuals perceive, interpret, and respond to risk and failure across different contexts. After identifying the six elements, the respondents explained each in detail. If their element did not fit the assigned categories, they were asked to think of an alternative one with a better fit.

#### 3.2.2 Elicitation of constructs

They were then presented with triads: three selected elements (see asterisks in Table 1) out of the six elements, in an iterative process. Participants were asked to compare and contrast the three elements using the triad question:

**TABLE 1:** Repertory grid interview‐based result from informant 3.

		Elements						
	Construct pole (score 1)	a	b	c	d	e	f	Opposite construct pole (score 5)
1	Not following process or planned mitigations	*		*		*		Followed process or planned mitigations
		1	3	3	5	2	4	
2	Unique, innovative response	*		*			*	The boilerplate process followed in response
		5	2	4	5	3	1	
3	Hard skills response	*			*	*		Soft skills response
		2	3	3	1	5	4	
4	Weak leadership	*			*		*	Strong leadership
		2	3	3	1	5	4	
5	Single group working		*	*		*		Multi cross‐organizational working
		2	2	3	3	4	5	
6	Individual		*	*			*	Team
		1.5	1	3	4.5	5	4	
7								
8								
9								
10	Late activation of response	4	4	4	5	1	2	Early activation of response

“*Considering these three incidents, please think about how two were similar and thereby different from the third one regarding how you anticipated and responded to them?”*

To further clarify the meaning assigned by the participants to the elicited construct, the technique of “laddering down” (Jankowicz, 2004) was used, which involved probing questions such as: “What does the [construct] mean to you in regards to responding to this incident?” and “In what way is this [construct] vital to you in terms of responding to an [element]?” The laddering question was designed to explore participants’ understanding of the construct they used to compare and contrast three elements.

In addition to the elicited constructs, we provided an ascribed outcome construct of “Late Activation of Response” and “Early Activation of Response” to establish if their response to errors was activated early or late (see Table 1, iteration 10). As emphasized before, the ascribed outcome construct of early versus late response activation provides an important metric for evaluating organizational efficacy in HROs (Hopkins, 2009a; Roberts, 1990b, 2009; Vogus et al., 2014; Weick et al., 1999) in complex systems.

#### 3.2.3 The rating of elements

After eliciting the constructs, we asked participants to define two opposite extremes for each construct. They then rated all six elements on a scale of 1–5 based on their interpretation of these polar extremes. A rating of “1” represented elements close to the construct or emergent pole, and a rating of “5” represented elements best explained by the contrast or implicit pole. Table 1 shows a sample of a completed repertory grid with six elicited bipolar constructs and one ascribed construct.

To illustrate how to read the grid in Table 1, we can look at the first row of the grid, which contrasts the construct poles “Not following process or planned mitigations” with “Followed process or planned mitigations.” The scores assigned to each element (a–f) in this row indicate how closely they align with either of these extremes. For example, if an element scores 1, it is perceived as strongly aligned with “Not following process or planned mitigations.” Conversely, a score of 5 would suggest that the element is seen as closely aligned with “Followed process or planned mitigations.” Intermediate scores like 3 reflect a more balanced or neutral position between the poles.

### 3.3 Multiple grid data analysis

#### 3.3.1 Core categorization process

Based on 103 repertory grid interviews, we elicited a set of 618 elements or experiences of responding to errors comprising an equal number of near‐miss and accidents. We extracted 659 constructs, yielding an average of 6.4 constructs per interview. We conducted a core categorization procedure essential for distilling the complex perceptual data on personal constructs associated with responses to events categorized as near‐misses and accidents into categories. This core categorization procedure allowed us to classify and organize the personal constructs elicited from participants, thereby facilitating a deeper understanding of how individuals perceive their world. By categorizing these constructs, we identified core response categories (Jankowicz, 2004).

The core categorization and reliability process (see Table 2) is structured into initial coding, recalibration, and validation. In the first step, initial coding, the team began with initial coding where two separate teams (Team 1: researchers A and B, and Team 2: researchers C and D) independently categorized all constructs. Team 1 identified 24 categories during this step, while Team 2 identified 18. After comparing their results, we agreed on 23 common constructs. However, the intercoder reliability at this stage was relatively low, at 56%, indicating moderate agreement between the teams.

**TABLE 2:** Core categorization and reliability process.

	Core categorization and reliability process		
Step	Step 1	Step 2	Step 3
Measure	Initial coding	Recalibration	Validation
Process	Initial categorization	Word clouds	Reliability table
		Enhanced categorization	Word clouds
			Construct cards
			Pareto analysis
Number of categories	Team 1 (A and B): 24	Team 1 (A and B): 18	Independent researcher E
	Team 2 (C and D): 18	Team 2 (C and D): 17	
Agreed to common constructs	23	18	13
Intercoder reliability	56%	68%	76%

In the second step, recalibration, both teams worked to enhance their categorization by incorporating an additional technique called NVivo word clouds. As a result, Team 1 reduced its categories to 18 and Team 2 to 17. At this point, the two teams reached an agreement on 18 common constructs, and the intercoder reliability improved to 68%, demonstrating better consistency and agreement between the two teams.

The final step, validation, involved an independent review conducted by Researcher E to ensure the robustness of the categorization. After this independent review, a total of 13 constructs were agreed upon. This final step resulted in the highest intercoder reliability of 76%, reflecting a strong level of agreement after the categorization process had been validated and refined.

#### 3.3.2 Unique frequency analysis

Our data analysis established the frequency of constructs using unique frequency (UF) analysis. This method assesses whether a construct mentioned by one participant also appears in others’ responses, with the percentage indicating how widely a construct is shared among respondents. For this research, we set a UF cut‐off point of 25%, meaning a construct had to be mentioned by at least 25% of participants (26 or more out of 103 interviewees) to be considered significant. This threshold, which is higher than the more common 10%–20% range typically used in repertory grid studies (Fransella & Bannister, 2004; Jankowicz, 2004) was chosen to ensure that only the most prevalent and potentially impactful constructs were included in our final analysis. Table 3 lists the construct categories identified through this %UF approach, with eight out of thirteen categories meeting this higher criterion, underscoring their relevance and commonality across the study's cohort.

**TABLE 3:** Common construct categories (in descending order of frequencies) (Y—Yes; N—No).

Construct category	Illustrative quotes	Construct pole definition	Opposite construct pole definition	Freq. total	Unique freq. (>26)
Following	*“We had procedures; we instantly knew what to do…”*	Following procedures, processes, or policies.^a^	Ignoring, delaying, or innovating a response.	68	Y
Simplifying	*“It was complicated; trying to resolve it was not simple.”*	Rendering a problem or solution, or response as complex, complicated, nonroutine, or time‐sensitive.	Simplifying a problem or solution, or response as simple or small, routine or nonurgent.	46	Y
Heeding	*“Completely unexpected, unplanned, not even, not even a risk identified against any of them.”*	Anticipating, planning, predicting, or having oversight of warnings or risks.	Ignoring or overlooking unpredictable, unknown, or unforeseeable warnings.	44	Y
Influencing	*“There was nothing we could do about that.”*	Recognizing the ability to influence and control responses or actions or solutions.	Accepting low influence over responses, actions, or solutions outside of control.	40	Y
Engaging	*“… primarily engaging with internal stakeholders only within the team.”*	Engaging, involving, or motivating stakeholders.	Ignoring disinterested, opposed, or demotivated stakeholders.	34	Y
Aligning	*“… developing the bigger picture around them and justifying the decisions.”*	Individual or single‐handed or noncoordinated working or response.	Cross‐organizational or cross‐team collaborative or coordinated response.	33	Y
Clarifying	*“It was evident what we had to do.”*	Seeking to understand, clarify, prioritize, or measure aims, objectives, or direction.	Appreciating aims, objectives, or outcomes as unclear or not understood.	28	Y
Communicating	*“Lack of information or the wrong information on both of them; people were telling us different things.”*	Sharing information extensively, transparently, or openly.	Hiding or missing off or nondisclosing information.	27	Y
Deferring	*“…responding in terms of people and training; expertise to pour over data and understand the data they are looking at.”*	Resigning to incompetence or lack of skills or experience or professionalism.	Yielding to skills, knowledge, or experience.	25	N
Accessing	*“I had access to everyone I needed to speak to resolve it.”*	Relying on internal support or resources.	Accessing external support.	23	N
Evaluating	*“It took quite a while to investigate where it had come from, what it was.”*	Lack of investigating, reviewing, or analyzing.	Investigating, reviewing, or analyzing meaningfully, frankly, or with attention to detail.	15	N
Leading	*“Strong, clear leadership …”*	Weak or poor leading.	Strong directing.	7	N
Learning	*“The policy was changed to prevent it from happening again.”*	Lack of learning and change in the aftermath of an incident.	Learning and changing in the aftermath of an incident.	6	N

^a^ Informed by NVivo Word clouds.

#### 3.3.3 Honey's content analysis

Honey's content analysis (Honey, 1979; Rojon et al., 2018) is a quantitative analytical method developed to assess how individuals conceptualize and evaluate constructs, with a particular focus on cognitive complexity and interpretive depth. Rooted in cognitive psychology and constructivist learning theories, Honey's content analysis was originally designed to measure the richness and structure of individuals’ conceptual understanding. The method builds on Kelly's Personal Construct Theory (1955), which emphasizes that people interpret the world through individualized mental frameworks shaped by experience, learning, and social context.

This method systematically quantifies qualitative data by analyzing the language and structure of participants’ responses, identifying patterns in their reasoning processes. By mapping these patterns, the Honey's content analysis enables researchers to assess the sophistication of conceptual thinking, distinguishing between simple, fragmented understandings and more integrated, nuanced perspectives. This makes it particularly useful in fields such as education, psychology, and risk management, where it is important to gauge how individuals engage with complex or abstract ideas.

The essence of the Honey's content analysis, as highlighted by Easterby‐Smith (1980) and Raja et al. (2013) in this study, is to determine how the poles of the elicited construct categories are associated with the ascribed bipolar construct of early and late activation in response to near‐misses and accidents.

The Honey's content analysis uses two key metrics: the mean percent similarity score and the H–I–L score. The mean percent similarity score as an index of adaptive capacity *within* constructs reflects how closely participants align their responses with constructs related to the early activation of a response. A high mean percent similarity score (70% or above, as shown in Table 3) suggests that participants strongly prefer the construct pole. Scores between 60% and 70% indicate a moderate preference for this pole. A similarity score between 50% and 60% suggests a balanced preference, with participants being relatively evenly distributed between the construct and its opposite pole. A midpoint score—neither high nor low—shows a balanced use of both contrasting poles within a construct. It indicates a both/and mindset, demonstrating an ability to engage with both poles flexibly. This balance in interpretation represents a higher adaptive capacity within constructs, where participants can fluidly apply constructs in diverse scenarios without rigid preferences for early or late response actions.

The H–I–L score categorizes responses into High (H), Intermediate (I), and Low (L) levels of relevance, reflecting how participants prioritize constructs in early activating a response to near‐misses and accidents. The highest percentage score on H–I–L index determines whether the construct falls under the High, Intermediate, or Low level of relevance. Low to Intermediate scores across constructs indicate that no single construct dominates, signaling a more balanced distribution of relevance among constructs. This suggest a higher adaptive capacity *between* constructs, as participants are not overly reliant on any one construct, enabling a flexible, context‐sensitive approach to managing incidents. In contrast, a distribution that consists of more High scores would suggest that participants overemphasize certain constructs, potentially limiting their adaptive range and, consequently, their adaptive capacity between constructs.

### 3.4 Content validity, reliability, and generalizability

#### 3.4.1 Content validity

**FIGURE 1:**
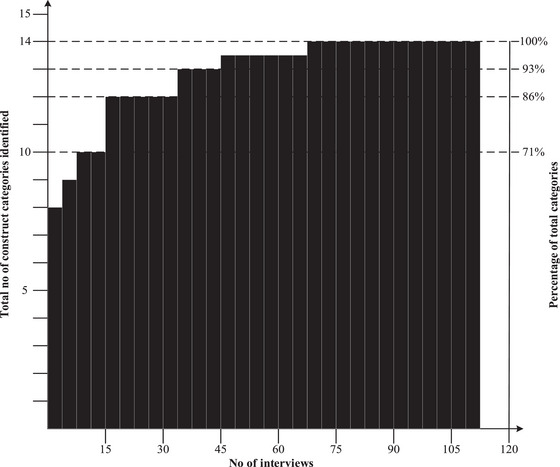
Results of the Pareto analysis.

Using the Pareto analysis we established the point of data saturation (see Figure 1) during the research process (Goffin & Koners, 2011). Data saturation is when no additional categories emerge from the core categorization process. This technique highlights when further data collection may no longer contribute new insights to the research findings.

The Pareto analysis shows that the first 15 interviews defined ten construct categories (71% of all classified construct categories). The following seven interviews resulted in the identification of 86% of all construct categories. At the 45th interview, 93% of all classified construct categories were already identified. Finally, 100% of all construct categories were determined after 65 interviews. We carried out a further 43 interviews to validate the development of all construct categories (Table 3).

#### 3.4.2 Generalizability

Given that the respondents in our study selected their elements, it is important to acknowledge the influence of their contexts on the constructs elicited (Adams‐Webber, 2001; Bell, 2004; Fransella & Bannister, 2004; Kelly, 1995; Neimeyer, 1993; Stewart et al., 1981). Since participants selected their own elements, the constructs they articulated reflect their unique cognitive frameworks, often extending beyond the immediate situational factors associated with those elements. This means that despite the research being framed within the specific operational environment of defense procurement, the constructs elicited reflect deeper cognitive patterns and experiences. This suggests that the insights gained from these constructs could be applied to a broader range of contexts and incidents, extending their relevance beyond defense procurement projects (see limitations). Thus, the constructs carry potential generalizability, as they are not solely shaped by the immediate situational variables associated with the elements chosen by each respondent.

## 4 RESULTS

### 4.1 Adaptive range

In our analysis of categories of corrective actions toward near‐misses and accidents, we identified eight categories of actions that participants deemed salient based on the %UF analysis. Table 3, in descending order of the UF, shows that the construct categories of *Following*, *Simplifying*, *Heeding*, *Influencing*, *Engaging*, *Aligning*, *Clarifying*, and *Communicating* have met the threshold of %UF, so they are deemed salient by the respondents. These construct categories are significant in the participants’ cognitive representations of responding to near‐miss and accidents. The *Deferring*, *Accessing*, *Evaluating*, *Leading*, and *Learning construct categories* failed to meet the threshold and were deemed insufficiently salient as construct categories requiring further analysis.

The adaptive range of the bipolar construct categories, meeting the %UF threshold, illustrates the spectrum of possible responses that can be adapted to events categorized as near‐miss errors and accident failures.

1. *Following* (vs. *Deviating*): This construct represents the range between strictly adhering to established procedures, processes, or policies and opting to ignore, delay, or innovate responses. On one hand, following established guidelines ensures consistency and predictability in actions. Conversely, choosing to ignore or delay actions allows for a more flexible, creative approach, fostering innovation and adaptability in situations where standard procedures may not apply or when novel solutions are necessary.
2. *Simplifying* (vs. *Complicating*): This construct encompasses the ability to simplify a problem, solution, or response, rendering it straightforward, routine, or nonurgent, versus viewing it as complex, complicated, nonroutine, or time‐sensitive. The ability to simplify enables quick decision‐making and action in scenarios where efficiency is key. Conversely, recognizing and addressing complexity is essential in situations that require nuanced understanding and comprehensive problem‐solving.
3. *Heeding* (vs. *Ignoring* and *Overlooking*): This construct describes a spectrum ranging from actively anticipating, planning, or predicting risks and having oversight of potential warnings to ignoring or overlooking unpredictable, unknown, or unforeseeable warnings.
4. *Influencing* (vs. *Accepting*): This construct represents the range between recognizing the ability to influence and control responses, actions, or solutions and accepting low influence over events outside one's control. On one hand, acknowledging influence enables proactive engagement and intervention, while on the other, accepting low influence emphasizes adaptation to external conditions and a focus on areas within one's control.
5. *Engaging* (vs. *Disregarding*): This construct covers the range from actively engaging, involving, or motivating stakeholders to ignoring, disengaging from, or dealing with disinterested, opposed, or demotivated stakeholders. Engagement may be salient for collaboration and collective action, while disengagement can be necessary when stakeholder involvement is minimal or counterproductive.
6. *Aligning* (vs. *Working Individually and Noncoordinated*): This construct describes the spectrum between aligning responses in a cross‐organizational or cross‐team collaborative manner and operating individually or in a noncoordinated way. Alignment may foster unity and coordinated efforts, which is beneficial in complex situations requiring diverse expertise. In contrast, individual or noncoordinated actions may allow more autonomous responses when coordination may slow down the process.
7. *Clarifying* (vs. *Appreciating*): This construct captures the range between seeking to understand, clarify, prioritize, or measure aims, objectives, or directions and appreciating or tolerating ambiguity where aims or outcomes are unclear. Clarification may be important for focused and goal‐directed action, whereas appreciating ambiguity allows flexibility and adaptability in uncertain situations where not all information is available.
8. *Communicating* (vs. *Nondisclosing*): This construct reflects the range between extensively sharing information transparently and openly versus hiding, missing, or nondisclosing information. Effective communication supports transparency and shared understanding, while nondisclosure may be strategic to manage sensitive information or prevent unnecessary alarm.

Each of these constructs represents a bipolar range of responses, allowing for a wide range of adaptive responses based on the specific demands of the situation. Shifting along these spectrums enables individuals and organizations to navigate complexity and uncertainty more effectively, adapting their approaches to meet varying challenges.

### 4.2 Adaptive capacity

The H–I–L classification (High–Intermediate–Low) provides further insight into how participants prioritize constructs when responding to incidents. The H score (High relevance) represents constructs that participants consider crucial in early response activation, meaning they strongly associate those constructs with an effective incident response. An I score (Intermediate relevance) suggests that participants view the construct as relevant but not dominant, allowing for flexibility in its application. Finally, an L score (Low relevance) indicates that participants do not consistently rely on the construct in their response strategies.

When constructs show a greater spread across I and L scores (see Table 4), rather than being concentrated in H, it suggests that participants adopt a more balanced approach, utilizing a wider range of responses rather than fixating on specific constructs. This distribution of relevance allows for greater adaptability, as participants are able to fluidly engage with different constructs depending on the incident context. However, when constructs are concentrated in the H category, it suggests a rigid approach (less adaptive capacity), where participants repeatedly fall back on the same constructs, potentially reducing their ability to navigate complex, uncertain situations effectively.

**TABLE 4:** Adaptive capacity of responses to events construed as near‐miss errors (in descending order of degree of relevance).

Construct category	Construct pole	Mean %‐similarity score	Opposite construct pole	Mean %‐similarity score	Preference (toward the construct pole)	H%	I%	L%
Influencing	Recognizing the ability to influence and control responses or actions or solutions.	71.94	Accepting low influence over responses, actions, or solutions outside of control.	28.06	Strong preference	40.00	30.00	30.00
Following	Following procedures, processes, or policies.	63.23	Ignoring, delaying, or innovating a response.	36.77	Moderate preference	38.67	24.00	37.33
Communicating	Sharing information extensively, transparently, or openly.	79.66	Hiding or missing off or nondisclosing information.	20.34	Strong preference	34.48	51.72	13.79
Simplifying	Rendering a problem or solution, or response as complex, complicated, nonroutine, or time‐sensitive.	61.64	Simplifying a problem or solution, or response as simple or small, routine or nonurgent.	38.36	Moderate preference	26.23	49.18	24.59
Aligning	Cross‐organizational or cross‐team collaborative or coordinated response. Working or response.	50.47	Individual single‐handed or noncoordinated	49.53	Balanced preference	20.69	48.28	31.03
Heeding	Anticipating planning or predicting, or having oversight of warnings or risks.	66.66	Ignoring or overlooking unpredictable, unknown, or unforeseeable warnings.	33.34	Moderate preference	25.00	45.45	29.55
Engaging	Engaging, involving, or motivating stakeholders.	52.73	Ignoring disinterested, opposed, or demotivated stakeholders.	47.27	Balanced preference	9.09	39.39	51.52
Clarifying	Seeking to understand, clarify, prioritize, or measure aims, objectives, or direction.	57.98	Appreciating aims, objectives, or outcomes as unclear or not understood.	42.02	Balanced preference	26.09	34.78	39.13

Honey score high (H% > I% and L%): High relevance.

Honey score balanced (I% > H% and L%); Moderate relevance.

Honey score low (L% > H% and/or I%); Low relevance.

For the *Influencing* construct, defined as recognizing the ability to influence and control responses or actions, the mean percent similarity score is 71.94%, indicating a strong alignment with the construct of early activation. This suggests that participants strongly prefer to activate responses based on their perceived ability to control or influence the situation when responding early. The H–I–L score reveals that 40% of participants rated this construct highly relevant (H), meaning that influence is critical for early responses to near‐misses and accidents. However, 30% rated it as having intermediate (I) relevance, indicating that for some, influencing is also seen as helpful in both early and delayed responses. Another 30% rated it as having low (L) relevance, suggesting that, for a smaller group, influencing may not be as relevant in early activating a response.

For the *Following* construct, which pertains to following procedures, processes, or policies, the mean percent similarity score is 63.23%, showing a moderate preference for adherence to established guidelines during incident management. The H–I–L score reflects this mixed perception: 38.67% of participants rated this construct as highly relevant (H) for early responses, showing that many participants value following procedures when responding to incidents. However, 24% rated it as having intermediate relevance (I), indicating that procedures are sometimes balanced with other approaches during response activation, and 37.33% rated it as having low relevance (L). This sizable proportion suggests that, in some cases, participants may prioritize improvisation or delayed adherence to formal procedures, especially in rapidly changing or unpredictable scenarios. This more moderate preference indicates that while following processes is important, there is also significant flexibility in adapting beyond standard protocols in response to near‐miss errors.

Regarding events construed as accident failures (see Table 5), *Aligning* in the context of incident management, particularly for accidents, reveals some important insights into participants’ perceptions of cross‐organizational or cross‐team coordination. The mean percent similarity score for *Aligning* is 58.11%, indicating a relatively balanced preference, with a moderate inclination toward individual or single‐handed responses over fully coordinated efforts across teams or organizations. This suggests that participants slightly favor more independent approaches to managing incidents in early activating a response but still recognize the importance of collaboration.

**TABLE 5:** Adaptive capacity of responses to events construed as accident failures (in descending order of relevance).

Construct category	Construct pole	Mean %‐ similarity score	Opposite construct pole	Mean %‐similarity score	Preference (toward the construct pole)	H%	I%	L%
Aligning	Individual or single‐handed or noncoordinated working or response.	58.11	Cross‐organizational or cross‐team collaborative or coordinated response.	41.89	Balanced preference	65.52	27.59	6.90
Clarifying	Seeking to understand, clarify, prioritize, or measure aims, objectives, or direction.	58.24	Appreciating aims, objectives, or outcomes as unclear or not understood.	41.76	Balanced preference	13.04	60.87	26.09
Communicating	Sharing information extensively, transparently, or openly.	54.63	Hiding or missing off or nondisclosing information.	45.37	Balanced preference	17.24	58.62	24.14
Simplifying	Rendering a problem or solution, or response as complex, complicated, nonroutine, or time‐sensitive.	52.91	Simplifying a problem, solution, or response as simple, minor, routine, or nonurgent.	47.09	Balanced preference	14.75	49.18	36.07
Engaging	Engaging, involving, or motivating stakeholders.	50.32	Ignoring disinterested, opposed, or demotivated stakeholders.	49.68	Balanced preference	21.21	42.42	36.36
Following	Following procedures, processes, or policies.	60.79	Ignoring, delaying, or innovating a response.	39.21	Moderate preference	12.00	54.67	33.33
Influencing	Recognizing the ability to influence and control responses or actions or solutions.	51.96	Accepting low influence over responses, actions, or solutions outside of control.	48.04	Balanced preference	35.00	40.00	25.00
Heeding	Anticipating planning or predicting, or having oversight of warnings or risks.	50.29	Ignoring or overlooking unpredictable, unknown, or unforeseeable warnings.	49.71	Balanced preference	25.00	36.36	38.64

Honey score high (H% > I% and L%): High relevance.

Honey score intermediate (I% > H% and L%); Moderate Relevance.

Honey score low (L% > H% and/or I%); Low Relevance.

The H–I–L scores for this construct offer further clarity. A significant 65.52% of participants rated *Aligning* as highly relevant (H), which indicates that despite the moderate preference for individual actions, many participants see cross‐team or cross‐organizational coordination as crucial for early response activation in accidents. This high level of relevance underscores the importance of collaboration in managing complex incidents where individual actions may not suffice.

On the other hand, 27.59% of participants rated *Aligning* as having intermediate relevance (I), suggesting that while collaboration is important, it may not always be necessary for every incident. For some scenarios, participants might perceive that a mix of individual initiative and coordination is needed, depending on the nature and complexity of the situation. Finally, only 6.90% rated this construct as having low relevance (L), meaning that very few participants dismiss the value of collaboration entirely when responding to accidents. This low percentage reflects a strong consensus on the overall relevance of teamwork in addressing and managing accident scenarios.

Table 6, a comparison of adaptive capacity, shows that for near‐miss situations, two relevant construct categories align with the early activation of a response: *Following* and *Influencing*. In an incident categorized as a near miss, the *Following* construct category plays a significant role in initiating a response early, with moderate adaptive capacity (see Table 6). This suggests that, although there is room for innovation and flexibility, the response remains moderately anchored to following procedures, processes, or policies. Meanwhile, the construct of *Influencing*, which involves recognizing the ability to control or shape responses, shows a low adaptive capacity in near‐misses. This implies that, during the early stages of responding to a near miss, there is a strong focus on recognizing how to influence or alter the course of events.

**TABLE 6:** Comparison of adaptive capacity.

Construct category	Construct pole	Opposite construct pole	Adaptive capacity near miss	Adaptive capacity accident failure
Engaging	Engaging, involving, or motivating stakeholders.	Ignoring disinterested, opposed, or demotivated stakeholders.	High	High
Aligning	Cross‐organizational or cross‐team collaborative or coordinated response.	Individual single‐handed or noncoordinated	High	High
Clarifying	Seeking to understand, clarify, prioritize, or measure aims, objectives, or direction.	Appreciating aims, objectives, or outcomes as unclear or not understood.	High	High
Simplifying	Rendering a problem or solution, or response as complex, complicated, nonroutine, or time‐sensitive.	Simplifying a problem or solution, or response as simple or small, routine or nonurgent.	Moderate	High
Heeding	Anticipating planning or predicting, or having oversight of warnings or risks.	Ignoring or overlooking unpredictable, unknown, or unforeseeable warnings.	Moderate	High
Following	Following procedures, processes, or policies.	Ignoring, delaying, or innovating a response.	Moderate	Moderate
Influencing	Recognizing the ability to influence and control responses or actions or solutions.	Accepting low influence over responses, actions, or solutions outside of control.	Low	High
Communicating	Sharing information extensively, transparently, or openly.	Hiding or missing off or nondisclosing information.	Low	High

In contrast, when examining accident failures, *Aligning* is the construct category that aligns with early response activation (see Table 5). In the case of an accident, the adaptive capacity of this construct is high (see Table 6), underscoring its importance for middle managers to embrace both poles of the spectrum. The need for alignment is much more pronounced in accident scenarios than near‐misses, as the situation's complexity may require a more dynamic approach toward collaborative working instead of single‐handed actions.

Overall, our study found a higher level of adaptive capacity in response to accidents compared to near‐miss errors. This increased adaptive capacity reflects a more significant potential to switch between or balance the two poles of a construct. For example, in the *Simplifying* construct, accidents show a higher adaptive capacity than near‐misses. In accident scenarios, there is a need to balance between rendering a problem as complex and time‐sensitive while also simplifying certain aspects to manage the situation effectively. This demonstrates the capacity to move between these two poles as necessary, which is less critical in near‐miss situations where the need to simplify may not be as urgent.

Similarly, accidents in the *Influencing* construct show a much higher adaptive capacity than near‐misses. During an accident, the prospect of influencing responses while acknowledging circumstances outside of one's control is high. This capacity to operate on both ends—asserting influence where possible and accepting limitations where necessary—appears salient for a resilient response.

In *Communicating*, accidents involve a higher capacity to manage both ends of the spectrum: sharing information openly while knowing when specific details need to be withheld. The key to adaptive capacity lies in the ability to navigate these tensions fluidly rather than rigidly adhering to one pole of a construct. In contrast, near‐miss situations favor one pole over the other, with less need for balancing conflicting demands.

## 5 DISCUSSION

This research addresses a significant gap in the existing literature on HROs by shifting the focus from observable practices and outcomes to the subjective and cognitive processes that underlie decision‐making. Rather than merely documenting what HROs do to achieve high reliability, this study delves into how organizational members construct and interpret their experiences and actions in response to various incidents, providing a deeper, more holistic understanding of how reliability is construed. By examining the interpretations of responses to near‐misses and accidents, this research uncovers the nuanced ways in which individuals within HROs perceive, analyze, and respond to different types of incidents. This approach acknowledges that reliability is not just a set of practices but is deeply rooted in the beliefs, perceptions, and mental models of those within the organization (S. Dekker, 2011; Reason, 2008; Weick, 1993; Weick et al., 2005).

Furthermore, this research highlights the dynamic and evolving nature of construed responses, emphasizing that these responses are not static but demonstrate an inherent adaptive capacity. This adaptive capacity serves as a foundation for continuously shaping both anticipatory and retrospective adaptations to incidents categorized as near‐misses and accidents (S. Dekker, 2011; Hollnagel et al., 2006; Reason, 1997; Roe & Schulman, 2008; Sutcliffe & Vogus, 2003; Weick & Sutcliffe, 2015). Focusing on the cognitive and interpretive processes that drive decision‐making, this study shows how organizations learn from past experiences and anticipate future incidents, allowing them to respond more effectively to various scenarios. This continuous adaptation process is important for maintaining high reliability in complex and uncertain environments, as it enables organizations to refine their strategies and practices over time based on new information and changing conditions.

By exploring the interplay between construed responses and adaptive range and capacity, this research contributes to a more comprehensive understanding of HROs. It underscores the importance of examining how organizational members’ interpretations shape their ability to effectively manage incidents perceived as near‐miss errors and accident failures. This perspective encourages a reevaluation of HRO theory, moving beyond a static view of organizational practices to consider the dynamic, interpretive processes that underpin high reliability in practice.

### 5.1 The adaptive range in HRO incident management

The concept of adaptive range within HRO theory offers a nuanced perspective on how organizations navigate responses to incidents along bipolar spectrums of near‐miss errors and accident failures. Our study identified a broad adaptive range encompassing eight distinct response constructs. Each construct category reflects a spectrum of behaviors and decision‐making approaches, shedding light on the organizational capacity to adjust responses based on strategic and situational demands.

For instance, the construct of *Following* emphasizes the bipolar range between strict adherence to established processes and the necessity to delay, ignore, or innovate responses when existing procedures are insufficient. This bipolar dynamic highlights how organizations balance the stability of rules and the flexibility required in unprecedented situations (Hollnagel et al., 2006; Reason, 1997; Weick et al., 1999).

Similarly, the *Simplifying* construct category pertains to the ability to render complex situations as either urgent, nonroutine events or routine occurrences, thereby reducing the cognitive load during high‐pressure situations. This reflects the tension between recognizing incidents as unique or reducing them to manageable, standardized responses (Hutchins, 1995; Flin & O'Connor, 2001; Weick, 1995).

The contribution to HRO theory is further extended by understanding *Heeding* as a construct that ranges from anticipating and predicting risks to overlooking unforeseeable warnings. This bipolarity underscores the importance of an organization's foresight, or lack thereof, in risk mitigation efforts. The bipolar range in this category provides insights into how HROs handle uncertainty—either by meticulously planning for possible outcomes or by managing the consequences of being caught off guard (Hollnagel et al., 2006; Reason, 1997; Vaughan, 2016; Woods, 2011, 2006; Woods & Branlat, 2011).

*Influencing* and *Engaging* both add depth to the HRO discussion by illustrating how organizations either recognize their ability to control outcomes or accept situations where their influence is limited. This shift from high to low control offers a lens into the organization's decision‐making processes under pressure while addressing stakeholder engagement. HROs must often engage diverse and sometimes disinterested stakeholders to mitigate risks effectively; the ability to shift between inclusive collaboration and individual or isolated responses expands the theoretical understanding of resilience in high‐stakes environments (Rasmussen, 1997; Roberts, 1990; Sutcliffe & Vogus, 2003; Weick & Sutcliffe, 2015; Woods, 2006).

Finally, constructs like *Aligning*, *Clarifying*, and *Communicating* provide further layers of analysis by exploring organizational responses from cross‐team coordination to individualistic efforts, from clear, measurable objectives to ambiguous goals, and from transparent communication to withholding information. Each bipolar spectrum encapsulates the various ways in which HROs adapt to maintain operational integrity across a range of near‐miss errors and accident failures (Hollnagel et al., 2006; March et al., 1979; Rasmussen, 1997; Weick & Roberts, 1993; Woods & Branlat, 2011).

The theoretical contribution lies in articulating these adaptive ranges, offering a systematic exploration of how organizations balance structure and flexibility when responding to incidents of varying severity. Thus, this discussion not only enhances the HRO theory but also provides a framework for understanding how adaptive capacities in organizations can either prevent accidents or respond to failures when they occur, expanding the scope of resilience and reliability management.

### 5.2 The adaptive capacity in HRO incident management

The findings from the comparative analysis of responses to near‐miss errors and accident failures offer valuable theoretical contributions to the understanding of HROs by illustrating how strategies and actions shift depending on the nature and severity of the incident. These insights deepen the understanding of adaptive capacity (Weick & Roberts, 1993; Woods, 2006), showing how organizations dynamically adjust their approach based on whether they are dealing with a potential risk or a full‐blown failure.

One key contribution is the role of *Influencing* early response activation. The strong preference for exerting control for incidents construed as near‐misses, as indicated by the high percent similarity score, highlights how HROs prioritize taking control and acting early when incidents are still in their manageable stages (Flin & O'Connor, 2001; Reason, 1997; Weick et al., 1999; Woods & Cook, 2006). This demonstrates the importance of proactive intervention in preventing minor errors from escalating into more significant issues. In contrast, during accident failures, the reduced relevance of influencing suggests a shift away from individual control to a more collective and collaborative response. This shift implies that in more severe incidents, influencing may be less about individual actions and more about the collective coordination required to manage the situation. The findings contribute to a deeper understanding of how adaptive capacity operates in different stages of an incident, moving from proactive control in near‐misses to broader, team‐based efforts during accidents.

The role of *Following* also offers theoretical insight into how HROs balance structure with flexibility. In near‐miss situations, there is a clear emphasis on adhering to established protocols as one of the prominent early responses, which reflects the reliance on procedures designed to detect and manage early signs of failure. This focus on procedures helps prevent risks from escalating and ensures that minor issues are addressed systematically. However, *Following* is not the prevailing response in case of accident failures, perhaps because strict adherence to procedures can become limiting. Instead, the emphasis lies on versatility, which allows for improvisation and adaptation to respond effectively to evolving circumstances. This highlights the dual necessity in HROs for structured responses and the ability to adapt beyond established protocols when the situation demands (S. Dekker, 2011; Flin & O'Connor, 2017; Reason, 1997; Vaughan, 2016; Weick & Sutcliffe, 2015).

The findings around *Aligning* responses underscore the importance of coordination and collaboration in managing accidents. In accident failures, the high relevance of aligning across teams and organizations highlights that coordinated efforts are crucial when dealing with more complex, high‐stakes scenarios. While near‐misses might be effectively managed through individual actions or within smaller teams, accidents often require a broad, collective response that involves multiple stakeholders working together. This finding enhances the theoretical understanding of how HROs manage complex incidents by shifting from individual autonomy in near‐misses to broader collaborative efforts in accidents. The strong preference for aligning in accidents suggests that cross‐organizational coordination becomes more critical as incidents escalate in severity (Comfort, 2007; Hollnagel et al., 2006; Roberts, 1990b; Weick & Sutcliffe, 2015).

### 5.3 Cultivating adaptive structuration for enhanced HRO incident management

The findings around adaptive range and adaptive capacity suggest important practical implications for organizations, particularly in tightly coupled high‐risk environments where near‐misses and accidents demand timely, flexible, dynamic responses. Drawing on adaptive structuration (Farjoun, 2010; Giddens, 1984), organizations benefit from expanding managers’ adaptive range to help them effectively assess the relevance of constructs when responding to incidents. By broadening the range of responses available to managers, organizations can enhance their ability to dynamically shift between different strategies depending on the (construed) severity and complexity of incidents.

One practical implication is the need for organizations to continuously evolve their structures to support a more flexible approach to incident management. As the findings show, constructs like *Influencing* and *Following* procedures are crucial for near‐misses, where early control and adherence to protocols help prevent escalation. However, the importance of *Aligning*—or cross‐organizational coordination—becomes more pronounced during more severe incidents such as accidents. This shift requires organizations to adapt their structures, enabling managers to move beyond “either/or” thinking, where they feel constrained by rigid, procedure‐bound responses, and toward “both/and” thinking, where they balance structured protocols with flexible, collaborative frameworks (Farjoun, 2010; Weick & Sutcliffe, 2015). Adaptive structuration posits that organizations are not constrained by its existing rules and procedures, allowing them to modify these structures based on real‐time improvisation, learning, and the nature of the incident.

In practice, this means that organizations should train managers to recognize and adjust their responses depending on the evolving nature of the incident. Managers need to be equipped with the tools to assess when to adhere to procedures and when to improvise or collaborate across teams, drawing mindfully on the adaptive range and capacity of responses. By expanding their adaptive range, managers can become more adept at identifying the relevance of specific actions—whether exerting control in the early stages of a near‐miss or coordinating with multiple teams during an accident. Training programs should emphasize situational awareness and foster both “either/or” thinking, where managers distinguish between clear‐cut situations requiring rigid adherence to one pole on the adaptive range of a response category, and “both/and” thinking, where they must integrate bipolar extremes to enact mindful decision making (Farjoun, 2010; Weick & Sutcliffe, 2015).

Furthermore, organizations could encourage managers to dynamically move between structured and improvisational responses to enhance adaptive capacity. The findings suggest that a strong preference for *Influencing* during near‐misses shifts toward a need for *Aligning* during accidents, highlighting the importance of managers effectively navigating this spectrum. Mindful decision‐making hinges on managers’ ability to alternate between “either/or” decisions, for example, when strict protocol adherence is necessary, and “both/and” decisions when a blend of control and flexibility is called for. Organizations should create environments that support this dynamic movement, ensuring that a single response mode does not constrain managers but can fluidly transition between response strategies as incidents unfold. This includes fostering a culture of adaptability, where managers feel empowered to step outside procedural constraints when necessary while still recognizing the importance of structure in more routine situations (S. Dekker, 2011; Grote, 2009; Vaughan, 2016; Weick, 1993).

In practice, this entails developing incident management frameworks that allow flexibility to dynamically adapt within the adaptive range of response options and improvise when conditions demand it. Managers should be trained to follow protocols and recognize when innovation and real‐time adjustments are necessary. This could involve scenario‐based training that simulates both near‐miss and accident scenarios, allowing managers to practice their adaptive range and capacity to incidents construed as near‐miss errors and accident failures (Edmondson, 1996; Hollnagel et al., 2006; Vogus & Sutcliffe, 2007; Weick & Sutcliffe, 2015; Woods & Branlat, 2011).

## 6 CONCLUSIONS

This study addresses a significant gap in the literature by providing a unified and comparative analysis of how middle managers construe and implement responses to near‐misses and accident failures. Whereas previous research has typically treated these incidents in isolation, this study examines how individuals and organizations adaptively manage both types of incidents together. The study highlights the distinct, yet complementary strategies employed in managing near‐misses versus accidents by focusing on middle managers’ responses. It underscores the importance of adaptive capacity—the ability to fluidly shift between response strategies based on incidents’ evolving severity and conditions.

The findings reveal that adaptive capacity is fundamentally about managing the tension between opposing poles of constructs, such as simplification and complexity or control and acceptance. Adaptive range and capacity, paired with collective mindfulness, enables organizations to tailor their response strategies to the specific demands of both near‐miss and accident scenarios. This ability to embrace both extremes as needed—rather than preferring one over the other—emerges as a key characteristic of resilient incident management.

The study acknowledges several limitations, such as the thresholds used in the repertory grid study to analyze construct categories. Future research could expand these thresholds to capture a broader range of constructs, providing deeper insights into how managers respond to near‐misses and accidents. In addition, as the repertory grid methodology captures cognitive processes at a specific time, future studies should investigate whether these constructs remain stable as managers gain more experience with different incident categories. A cross‐sector analysis could also help identify whether specific adaptive capacities vary in prevalence across industries, such as health care, aviation, or finance.

In conclusion, this study underscores the critical role of adaptive capacity in incident management, illustrating how organizations must flexibly navigate the unique complexities posed by near‐misses and accidents. By embracing the full range of response options ‐balancing both poles of a construct—organizations can enhance their ability to learn, adapt, and evolve. This approach strengthens resilience and fosters a culture capable of handling various incident scenarios. Future research should further explore the factors that influence the adaptive range and capacity in incident response. Such insights will be valuable in shaping training programs, decision‐making frameworks, and policy development, strengthening organizational resilience and high reliability across diverse sectors.
